# Retraction Note: Multifunctional nanoagents for ultrasensitive imaging and photoactive killing of Gram-negative and Gram-positive bacteria

**DOI:** 10.1038/s41467-026-68401-8

**Published:** 2026-03-04

**Authors:** Jiali Tang, Binbin Chu, Jinhua Wang, Bin Song, Yuanyuan Su, Houyu Wang, Yao He

**Affiliations:** Laboratory of Nanoscale Biochemical Analysis, Institute of Functional Nano and Soft Materials (FUNSOM), Soochow University, Suzhou, 215123 China

Retraction to: *Nature Communications* 10.1038/s41467-019-12088-7, published online 06 September 2019

The Editors have retracted this article. After publication, concerns were raised regarding the data presented in Fig. 7, specifically:

- In Fig. 7b HeLa, Ce6 group 3 and SiNPs group 3 images appear to overlap;

- In Fig. 7b HeLa, GP-Ce6-SiNPs control, group 1 and group 2 images appear to overlap;

- In Fig. 7c, Heart GP-Ce6-SiNPs (–) and PBS (–) images apear to overlap;

- In Fig. 7c, Lung PBS -, PBS + and GP-Ce6-SiNPs + images appear to overlap.

The authors have issued a Correction^1^ to address some of these concerns. However, further checks by the Publisher identified additional concerns:

- In Fig. 7b ARPE, Ce6 group 2 and 5 images appear to overlap;

- In Fig. 7c, Kidney PBS + and GP-Ce6-SiNPs + images appear to overlap.

The Editors therefore no longer have confidence in the presented data.

Jiali Tang and Houyu Wang agree with this retraction. The other authors have not responded to any correspondence from the editor or publisher about this retraction.
